# Keeping blood vessels out of sight

**DOI:** 10.7554/eLife.00948

**Published:** 2013-06-18

**Authors:** Dawn Sim, Marcus Fruttiger

**Affiliations:** 1 **Dawn Sim** is at the UCL Institute of Ophthalmology, London, United Kingdomd.sim@ucl.ac.uk; 2 **Marcus Fruttiger** is at the UCL Institute of Ophthalmology, London, United Kingdomm.fruttiger@ucl.ac.uk

**Keywords:** metabolism, retinal vasculature, soluble VEGF receptor-1, vascular demarcation, transgenic model, ophthalmology, Human, Mouse

## Abstract

Researchers have identified a soluble receptor that prevents blood vessels forming in the outer retina—a process that can lead to blindness—by sequestering vascular endothelial growth factor.

**Related research article** Luo L, Uehara H, Olsen T, Das SK, Zhang X, Simonis JM, et al. 2013. Photoreceptor avascular privilege is shielded by soluble VEGF receptor-1. *eLife* **2**:e00324. doi: 10.7554/eLife.00324

**Image** Expression of vascular endothelial growth factor (green) and sFLT-1, a receptor that inhibits this factor (red), in different layers of the retina

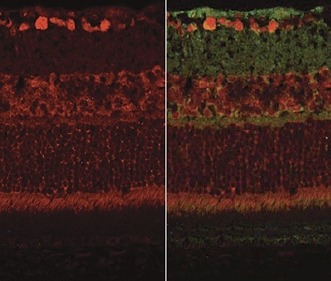

The tissues in our bodies are full of blood vessels, with the exception of cartilage and some parts of the eye. In particular, blood vessels are excluded from the cornea, lens and outer retina to prevent them from interfering with our ability to see. However, the outer retina is the most metabolically active tissue in the body, consuming more oxygen than even the brain, and the exclusion of blood vessels from this part of the retina has baffled vascular biologists for decades (Wangsa-Wirawan and Linsenmeier, 2003). Now, in *eLife*, Balamurali Ambati of the University of Utah and co-workers at various institutes in the US, Italy, Japan, Germany and China—including Ling Luo as first author—shed some light on this puzzle (Luo et al., 2013).

Metabolic demands are high in the outer retina because it contains large numbers of photoreceptor cells, which require a great deal of energy and oxygen to convert the light falling on the retina into electrical signals that can be processed by the brain. This can cause the outer retina’s oxygen levels to approach zero (Figure 1). Low oxygen levels normally stimulate the production of proteins, such as vascular endothelial growth factor (VEGF), that induce the formation of blood vessels and increase oxygen concentration. But this never occurs in the healthy retina, despite low oxygen levels, suggesting the presence of a powerful but unknown factor that can prevent blood-vessel formation.

**Figure 1.**
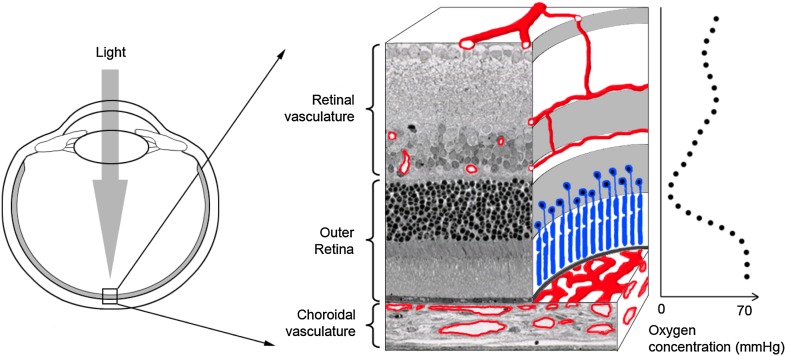
The outer retina is one of the few tissues in the body that do not contain blood vessels. Ambati and co-workers have now shown that a protein called sFLT-1 has a central role in preventing the growth of blood vessels in this part of the eye (Luo et al.). The two components of the outer retina are light-sensitive cells called photoreceptors (blue) and the retinal pigment epithelium (black line below photoreceptors). Blood and nutrients are supplied to the outer retina by two vascular networks (shown in red), the choroidal (below the outer retina) and the retinal vasculature (above). The concentration of oxygen approaches zero in the centre of the outer retina (see graph on right; Wangsa-Wirawan and Linsenmeier, 2003).

Luo et al. have now identified this mysterious factor as soluble fms-like tyrosine receptor (sFLT-1). This protein, which is a shorter version of a protein called FLT-1 (a full-length VEGF receptor), is known to inhibit the activity of VEGF in other tissues. Whereas FLT-1 is a membrane-bound protein that displays signalling activity, sFLT-1 is free to circulate in the blood and interact with VEGF. However, since it does not signal, it inhibits rather than enhances the activity of VEGF, and therefore suppresses the formation of blood vessels.

Ambati and co-workers have previously shown that sFLT-1 is responsible for excluding blood vessels from the cornea (Ambati et al., 2006). Now they have turned their attention to the back of the eye and discovered that the retinal photoreceptors and the retinal pigment epithelium can also produce sFLT-1. To test the function of sFLT-1 in the retina, they injected a neutralizing antibody into the subretinal space, the region between the photoreceptors and the retinal pigment epithelium (Figure 1), in a mouse model. This led to the invasion of blood vessels into the photoreceptor layer, as did an alternative blocking strategy in which RNA interference was used to knock down *sFlt-1* gene expression. Deletion of the entire *Flt-1* gene in photoreceptors or in the retinal pigment epithelium also caused blood vessels to grow into the outer retina, indicating that sFLT-1 directly prevents VEGF stimulating the formation of blood vessels.

These results are clinically relevant because the growth of blood vessels in the outer retina is a major cause of blindness. For example, in wet age-related macular degeneration (wet AMD), blood vessels from the choroid, which resides below the retinal pigment epithelium, breach that layer and invade the photoreceptors above (Figure 1). Luo et al. observed a reduction in sFLT-1 levels in the outer retina in post-mortem samples from patients suffering from wet AMD, raising the intriguing possibility that inadequate suppression of endogenous VEGF might contribute to AMD development in some proportion of patients.

Indeed, drugs that suppress the activity of VEGF in the eye have proven highly successful in treating wet AMD. The anti-VEGF agent Avastin is a humanized antibody that was initially approved for cancer therapy but soon found to dramatically improve vision in wet AMD patients. A modified version of this antibody (Lucentis) was subsequently approved for intraocular use (Rosenfeld et al., 2006), and a third, similar anti-VEGF agent (Eylea) has also recently been approved (Stewart et al., 2012).

Although these inhibitors have greatly improved treatment prospects for wet AMD and other types of visual loss—including diabetic retinopathy and other conditions that affect the working-age population—there is growing concern about the long-term use of anti-VEGFs because some base level of VEGF function appears integral to the health of the eye. The work of Luo et al. is reassuring, therefore, because it demonstrates that VEGF inhibitors occur naturally in the healthy retina, so it could be argued that treatment with anti-VEGF merely mimics what is already happening in the retina.

The challenges facing all anti-VEGF therapies include the need to block VEGF in specific layers of the retina and to avoid excessive concentrations of VEGF inhibitors in the eye. Gene therapy offers the possibility of sustained low-dose delivery, and trials that use adenoviral vectors to express *sFlt-1* in the subretinal space, arguably the most ‘natural’ anti-VEGF approach, are currently on-going with patients who suffer from wet AMD. Luo et al. also suggest that sFLT-1 expression by the retinal pigment epithelium is directional, which facilitates its export to the adjacent choroidal blood vessels. This would potentially allow the development of strategies to target anti-VEGF therapies more specifically within the eye.
